# Toxicity of BEP chemotherapy in germ cell tumors: focus on immune-mediated bleomycin pulmonary toxicity in comparison with EP and VIP regimens

**DOI:** 10.3389/fimmu.2026.1861834

**Published:** 2026-08-03

**Authors:** Patrik Olah, Peter Celec, Barbora Vlkova, Katarina Rejlekova

**Affiliations:** 1 2nd Department of Oncology, Faculty of Medicine, Comenius University and National Cancer Institute, Bratislava, Slovakia; 2 Institute of Molecular Biomedicine, Comenius University, Bratislava, Slovakia

**Keywords:** biomarkers, bleomycin, chemotherapy, germ cell tumors, pulmonary toxicity

## Abstract

Bleomycin, etoposide, and cisplatin (BEP) remains the cornerstone of curative first-line treatment for metastatic germ cell tumors (GCTs) across all prognostic risk groups. Although etoposide-cisplatin (EP) and etoposide-ifosfamide-cisplatin (VIP) are validated alternatives in selected clinical scenarios, oncologic efficacy among standard first-line cisplatin-based regimens is largely comparable within their respective indications. Consequently, treatment selection is increasingly driven by differences in toxicity profiles rather than antitumor effectiveness. Bleomycin exposure distinguishes BEP from other cisplatin-based regimens and underlies its distinct spectrum of adverse effects. Among these, pulmonary toxicity is the most clinically consequential and potentially irreversible complication. Bleomycin-induced pulmonary toxicity is characterized by marked interindividual variability, clinically significant toxicity occurring even at relatively low cumulative exposure despite cumulative dose remaining an important risk factor, and clinically meaningful morbidity and mortality in a predominantly young and otherwise curable population. Accumulating evidence indicates that bleomycin lung injury represents an immune-mediated process driven by dysregulated alveolar-immune crosstalk, innate immune activation, and profibrotic signaling. This review provides a toxicity-focused comparison of standard first-line cisplatin-based regimens used in the curative treatment of metastatic GCTs, particularly BEP, EP, and VIP regimens within the framework of the International Germ Cell Cancer Collaborative Group (IGCCCG) risk stratification and repositions bleomycin pulmonary toxicity as a model of immune-mediated chemotherapy toxicity. We integrate clinical and experimental data on immunopathogenesis and discuss emerging biomarkers of alveolar injury and immune activation, as promising investigational tools that require prospective validation before routine implementation in first-line treatment selection or pulmonary toxicity monitoring.

## 1 Introduction

Germ cell tumors (GCTs) represent one of the most curable solid malignancies in modern oncology, with long-term survival rates exceeding 80–90% even in the metastatic setting (1). This exceptional success has shifted clinical priorities from short-term disease control toward minimizing treatment-related toxicity and enhancing long-term survivorship, particularly in a young patient population with decades of life expectancy. In this context, standard first-line cisplatin-based chemotherapy regimens used in the curative treatment of metastatic GCTs remain of particular clinical importance, as differences in toxicity profiles may substantially influence individualized treatment selection despite comparable oncologic efficacy within specific prognostic groups.

Among cisplatin-based regimens, bleomycin, etoposide, and cisplatin (BEP) has long remained the backbone of curative first-line therapy for metastatic GCTs. First-line treatment selection is guided by prognostic risk stratification according to the IGCCCG (International Germ Cell Cancer Collaborative Group) classification (2, 3). In patients with good-risk disease, three cycles of BEP (BEP x3) represent the standard of care, while four cycles of etoposide and cisplatin (EP x4) provide a validated bleomycin-free alternative with equivalent oncologic efficacy (4, 5). In clinical practice, however, treatment selection is not based solely on IGCCCG risk stratification and may additionally reflect patient-specific risk factors for bleomycin-related pulmonary toxicity (6).

In contrast, for intermediate- and poor-risk disease, four cycles of BEP (BEP x4) remain the reference regimen, as EP has not demonstrated comparable efficacy (2, 7). The VIP (VIP x4) regimen (etoposide, ifosfamide, cisplatin) has demonstrated comparable oncologic efficacy to BEP as first-line treatment, but is associated with substantially increased hematologic toxicity (7). Therefore, the VIP regimen is primarily reserved as a bleomycin-sparing alternative for patients with contraindications to bleomycin (2, 7).

Given comparable cure rates within IGCCCG-defined first-line indications, regimen selection is increasingly driven by toxicity considerations. Bleomycin exposure represents the key differentiating factor between BEP and alternative regimens. While bleomycin causes several extrapulmonary adverse effects, pulmonary toxicity remains the most clinically consequential and potentially irreversible complication (8, 9).

Clinically overt bleomycin-induced pneumonitis occurs in approximately 10-20% of treated patients, with fatal outcomes reported in 1-3% (8, 10). Subclinical lung injury is substantially more frequent, with long-term impairment in diffusion capacity and persistent radiologic abnormalities documented years after treatment completion (10–12). Importantly, bleomycin pulmonary toxicity is unpredictable, does not correlate consistently with cumulative exposure, and demonstrates marked interindividual variability (8, 9).

Biologically, bleomycin-induced lung injury represents a prototypical model of chemotherapy-triggered, immune-mediated sterile inflammation. The lung is particularly susceptible because of low activity of bleomycin hydrolase, leading to prolonged tissue exposure (8). Subsequent oxidative epithelial and endothelial injury triggers innate immune activation, cytokine release, and profibrotic signaling that parallels mechanisms observed in inflammatory and fibrotic lung diseases (13, 14). Understanding these immunopathogenic mechanisms is essential for developing biomarker-guided strategies to prevent irreversible lung injury. Relevant literature was identified through PubMed searches and manual review of relevant references, with emphasis on clinically relevant studies and guideline-based evidence.

## 2 Clinical indications for BEP, EP, and VIP according to IGCCCG risk stratification

Prognostic stratification is fundamental to the management of metastatic GCTs and directly informs first-line treatment selection. The IGCCCG classification represents the most widely adopted prognostic model in clinical practice for metastatic GCTs, as it stratifies patients into good-, intermediate, and poor-risk groups based on primary tumor site, the presence of non-pulmonary visceral metastases, and pretreatment serum tumor marker levels (alpha-fetoprotein, human chorionic gonadotropin and lactate dehydrogenase). Patients classified as good-risk typically have gonadal or retroperitoneal primary tumors, absence of non-pulmonary visceral metastases and relatively low tumor marker levels, and achieve long-term cure rates exceeding 90% with cisplatin-based chemotherapy (2, 15, 16). In good-risk disease, three cycles of BEP (BEP x3) represent the preferred regimen. Four cycles of EP (EP x4) provide equivalent oncologic efficacy and are an accepted alternative in patients with contraindications to bleomycin or in those with heightened concern regarding pulmonary toxicity (4, 5). Beyond IGCCCG risk stratification, treatment selection in clinical practice may also be influenced by patient-specific factors associated with increased risk of bleomycin-related pulmonary toxicity, including advanced age, impaired renal function, chronic obstructive pulmonary disease, and reduced baseline pulmonary reserve (6).

Patients in intermediate- and poor risk groups are defined by higher tumor marker levels, unfavorable primary tumor sites (extragonadal, including mediastinal, pineal locations) or the presence of non-pulmonary visceral metastases and experience significantly worse prognoses compared with good-risk patients (2, 15, 16). In these patients, four cycles of BEP (BEP x4) remain the standard of care. EP has not demonstrated comparable efficacy and is therefore not recommended in these risk groups (2). The VIP regimen is reserved for patients with absolute or relative contraindications to bleomycin, including reduced or borderline renal function, age over 50 years, extensive pulmonary metastases, chronic obstructive pulmonary disease, or other pre-existing lung disease according to the NCCN guidelines and contemporary ASCO recommendations (6, 17). In addition, some centers preferentially administer VIP as first-line therapy in patients with primary mediastinal NSGCTs because of concerns regarding postoperative pulmonary complications following bleomycin exposure (17). Because VIP retains cisplatin as its therapeutic backbone and introduces ifosfamide, careful assessment of renal function, performance status and hydration feasibility remains essential before its use. Although oncologically comparable to BEP, its clinical use is limited by a substantially less favorable toxicity profile, with higher rates of severe myelosuppression (2, 7).

The presence of pulmonary metastases alone does not constitute a contraindication to bleomycin-containing chemotherapy. However, in a rare subgroup of poor-risk patients presenting with extensive pulmonary metastases accompanied by dyspnoea and/or hypoxaemia, abbreviated induction chemotherapy with cisplatin and etoposide followed by delayed bleomycin administration has been associated with a lower incidence of chemotherapy-related acute respiratory distress syndrome (ARDS) without compromising long-term outcomes (18). Once clinically stabilized, patients subsequently proceed to standard BEP, whereas VIP remains an appropriate bleomycin-sparing alternative when bleomycin cannot be safely reintroduced. Similarly, patients presenting with impending choriocarcinoma syndrome, characterized by widespread pulmonary metastases, markedly elevated hCG levels, and choriocarcinoma histology, may benefit from abbreviated induction chemotherapy with cisplatin and etoposide before initiation of standard curative-intent treatment, with subsequent reintroduction of bleomycin once clinically feasible (19).

## 3 Toxicity considerations in BEP, EP, and VIP

Despite a shared cisplatin backbone, BEP, EP, and VIP differ meaningfully in the nature and clinical relevance of treatment-related toxicity (**Table 1**). Cisplatin-associated nephrotoxicity, neurotoxicity, and ototoxicity occur across all regimens as class effects (8, 20). These toxicities are cumulative and largely predictable and only rarely necessitate premature discontinuation of curative-intent therapy, particularly in the setting of contemporary supportive care (20). Long-term cisplatin-associated complications, including persistent neurotoxicity, ototoxicity, renal impairment, cardiovascular disease, metabolic complications, hypogonadism, infertility, and secondary malignancies, are relevant across BEP, EP, and VIP and should be incorporated into survivorship counseling (21).

**Table 1:** Key toxicity characteristics of BEP, EP, and VIP (8, 10, 11, 13, 19–23).

Toxicity domain	BEP	EP	VIP
Bleomycin exposure	Yes	No	No
Severe neutropenia	Moderate	Moderate	High
Febrile neutropenia	~ 10-15%	~ 10-15%	~ 20-30%
Routine G-CSF	No	No	Yes
Supportive care requirements/mesna	No	No	Yes
Regimen-specific toxicity	Bleomycin-related pulmonary toxicity	None	Hemorrhagic cystitis, encephalopathy, renal tubular injury

BEP, bleomycin, etoposide, cisplatinl; EP, etoposide, cisplatin; VIP, etoposide, ifosfamide, cisplatin; G-CSF, granulocyte colony-stimulating factor.

Clinically relevant differences instead arise from regimen-specific components and treatment intensity. EP requires four cycles of therapy and may be associated with greater cumulative cisplatin exposure compared with BEP, potentially contributing to an increased burden of long-term cisplatin-related neurotoxicity and ototoxicity (22, 23). VIP avoids bleomycin but introduces substantial ifosfamide-related myelotoxicity. Consequently, patients treated with VIP face an increased risk of febrile neutropenia and infectious complications, requiring routine granulocyte colony-stimulating factor support to maintain treatment intensity (2, 7). Apart from hematologic toxicity, the incorporation of ifosfamide into VIP introduces additional supportive-care complexity and organ-specific adverse effects, particularly urothelial toxicity (e.g., hemorrhagic cystitis) requiring mesna prophylaxis and intensive hydration, as well as the potential for neurotoxicity (including encephalopathy) and renal tubular injury (24–26). In contrast, BEP avoids the profound hematologic toxicity characteristic of VIP but uniquely exposes patients to bleomycin-specific organ toxicity (7). Most notably, BEP carries a risk of bleomycin-related immune-mediated pulmonary toxicity, which represents the most biologically complex and clinically distinctive adverse effect among cisplatin-based regimens (8).

In this context, treatment-related toxicity extends beyond acute regimen selection and has important implications for long-term survivorship. The high cure rates achieved in metastatic testicular germ cell tumors have resulted in a population of long-term survivors who remain at risk for clinically significant late treatment-related toxicities. Survivors treated with cisplatin-based chemotherapy, particularly those exposed to higher cumulative cisplatin doses, may develop persistent neurotoxicity and ototoxicity, cardiovascular disease, secondary malignancies, pulmonary and renal impairment, hypogonadism, infertility, metabolic syndrome, and long-term reductions in quality of life. As they may emerge or persist decades after treatment, these findings underscore the importance of lifelong, structured survivorship care focused on the early identification, prevention, and management of late toxicities in germ cell tumor patients (21).

## 4 Pharmacology of bleomycin

**Figure 1:**
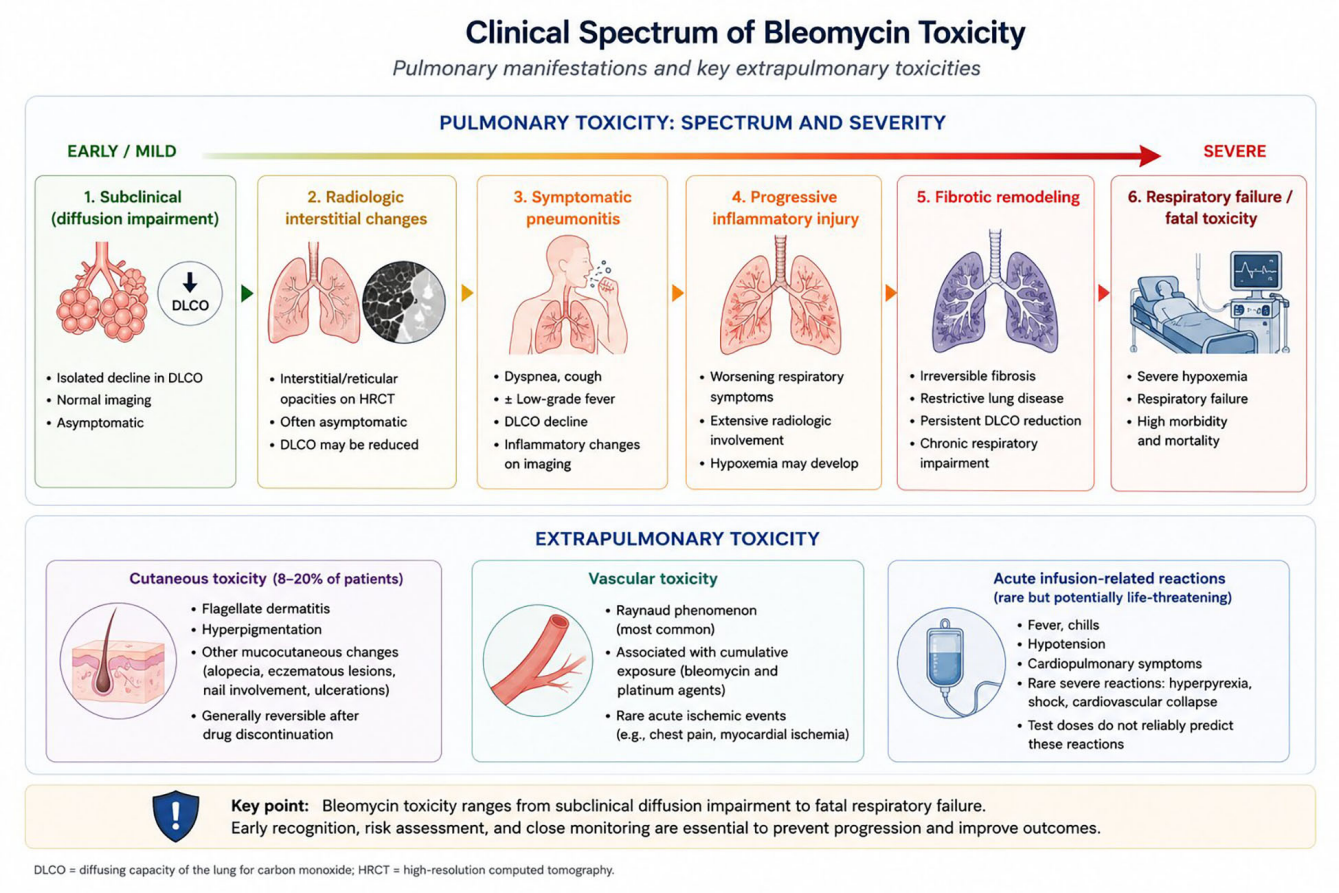
Clinical spectrum of bleomycin toxicity (Original content generated by AI) Schematic overview of pulmonary manifestations ranging from subclinical diffusion impairment to fibrotic remodeling and respiratory failure, with selected extrapulmonary toxicities associated with bleomycin exposure.

The antitumor activity of bleomycin is thought to derive from its capacity to generate free radicals, leading to oxidative damage of the deoxyribose backbone of DNA and subsequent single- and double-strand breaks. This DNA damage ultimately results in cell death of affected tumor cells. The generation of reactive oxygen species following bleomycin reduction is dependent on the ability of the drug to form complexes with divalent iron, a property that is central to its cytotoxic mechanism of action. The cytotoxic potential of bleomycin is further modulated by the activity of bleomycin hydrolase, an enzyme responsible for intracellular drug inactivation. Bleomycin hydrolase is expressed predominantly in tissues such as the liver, spleen, bone marrow, and intestinal epithelium, where it limits local drug accumulation and toxicity (27, 28). In contrast, minimal or absent enzymatic activity has been demonstrated in the skin and lungs, a finding that may explain the heightened susceptibility of these organs to bleomycin-induced toxic effects and provide a biologic basis for the characteristic pattern of pulmonary and cutaneous toxicity observed in clinical practice (**Figure 1**) (8, 28).

From a pharmacokinetic standpoint, bleomycin is characterized by a relatively simple but clinically relevant profile. Following intravenous administration, bleomycin exhibits a rapid biphasic decline in plasma concentrations, with a terminal elimination half-life of approximately 1.5–3 hours in patients with normal renal function. Bleomycin is eliminated predominantly by the kidneys, with approximately 50-70% of an administered dose excreted unchanged in the urine. Thus, kidney function-related differences contribute to systemic exposure and toxicity risk (27, 28). The pharmacokinetic characteristics of bleomycin provide a clinically relevant framework linking renal function to toxicity risk, particularly pulmonary toxicity, in the context of concurrent treatment with nephrotoxic agents such as cisplatin (8, 27, 28).

## 5 Bleomycin-related toxicity beyond the lung

Beyond the most well-known adverse effect of bleomycin, its administration is associated with numerous extrapulmonary adverse events, most prominently affecting the skin (8). In addition, rare but potentially life-threatening acute hypersensitivity or infusion-related reactions have also been reported during or shortly after bleomycin administration, with manifestations ranging from fever and chills to severe hyperpyrexia, hypotension, shock, and cardiovascular collapse (29–32). Bleomycin test doses have historically been used in an attempt to predict these reactions, however, available evidence does not clearly support their predictive value (33).

### 5.1 Cutaneous toxicity

Consistent with the tissue-specific susceptibility of bleomycin, cutaneous toxicity is a well-recognized manifestation of bleomycin exposure, with skin-related adverse effects reported in approximately 8–20% of treated patients. Beyond nonspecific mucocutaneous changes such as alopecia, eczematous lesions, nail involvement and ulcerations, bleomycin is characteristically associated with flagellate dermatitis and hyperpigmentation (34, 35). These cutaneous manifestations are generally reversible following drug discontinuation (36).

### 5.2 Vascular toxicity

Bleomycin-containing chemotherapy has been associated with vascular toxicity, most commonly Raynaud’s phenomenon. Raynaud-like symptoms are frequent in long-term survivors of testicular cancer treated with cisplatin-based regimens, affecting approximately one-third of patients, and have been reported to correlate with cumulative exposure to bleomycin and platinum agents as well as impaired quality of life and increased cardiovascular risk (27, 37). In addition, rare cases of acute ischemic events, including chest pain and myocardial ischemia occurring during bleomycin infusion, have also been described and warrant clinical awareness when evaluating cardiovascular symptoms during treatment (38).

### 5.3 Hepatic, renal, and neurologic considerations

Other bleomycin-related systemic toxicities are infrequently reported. Hepatic involvement is most often limited to mild, transient elevations of liver enzymes with return to baseline after cessation of therapy, while clinically significant hepatic injury appears to be rare and has been described primarily as isolated cases of inflammatory liver disease temporally linked to bleomycin exposure (39, 40). In patients treated with bleomycin-containing regimens such as BEP, renal dysfunction is common and largely driven by the cisplatin backbone, although it remains clinically relevant as renal function modulates bleomycin exposure (8, 41). Similarly, chronic neurotoxicity, such as tinnitus, hearing impairment, and paresthesia, observed in long-term testicular cancer survivors correlates predominantly with platinum exposure rather than bleomycin (42).

## 6 Bleomycin-induced pulmonary toxicity

Pulmonary toxicity represents the most clinically consequential adverse effect of bleomycin and the principal reason for treatment modification or avoidance (8). Bleomycin-induced lung injury is characterized by marked interindividual variability, and clinically relevant cumulative dose dependence, although clinically significant toxicity may also develop in patients without identifiable risk factors (8, 11, 43). Clinical outcomes are heterogeneous, ranging from gradual normalization of pulmonary symptoms and functional abnormalities following bleomycin discontinuation, with or without corticosteroid therapy, to irreversible pulmonary fibrosis (8, 43). The biological basis for these clinical observations is discussed below.

### 6.1 Immunopathogenesis of bleomycin-induced pulmonary toxicity

**Figure 2:**
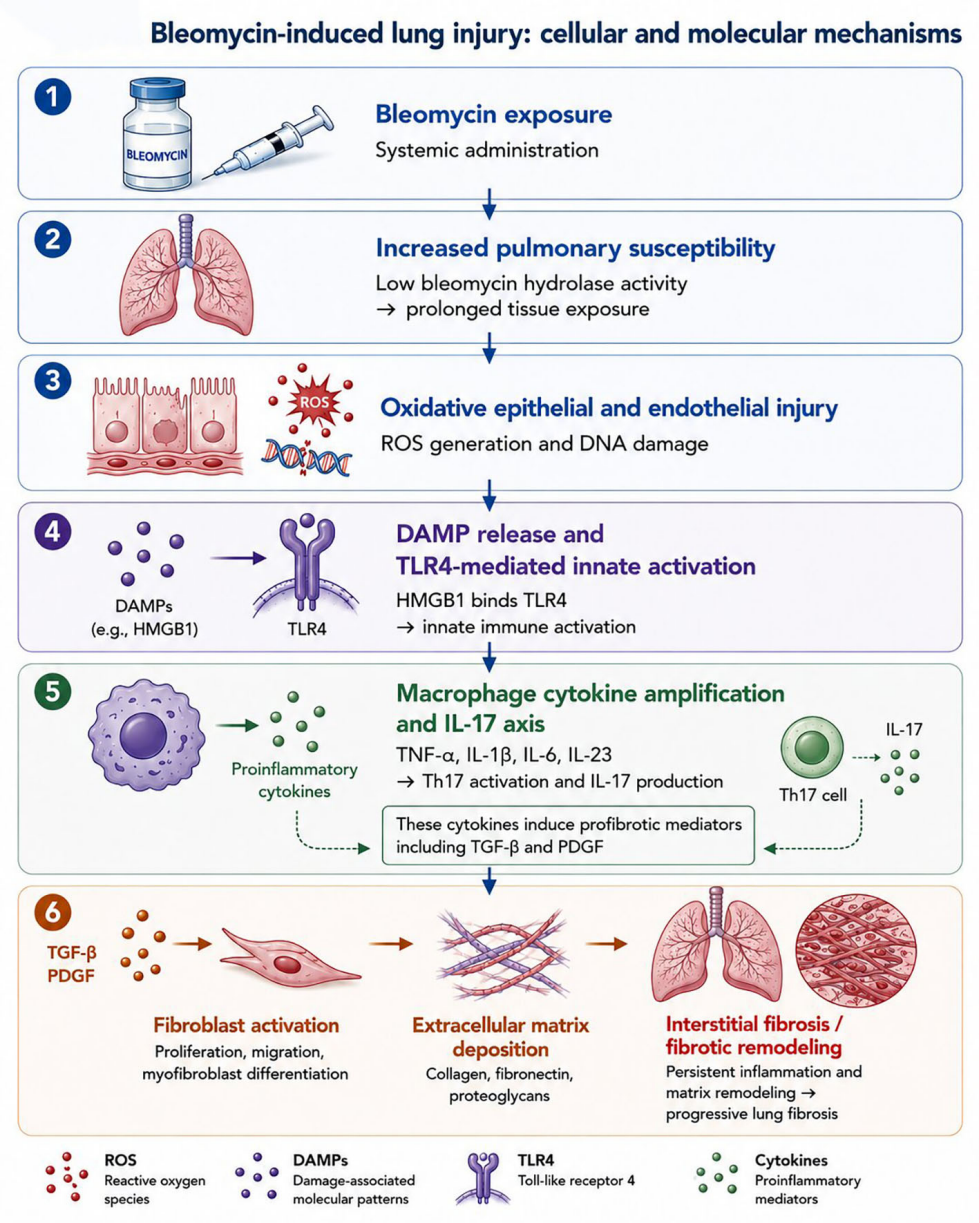
Bleomycin-induced lung injury: cellular and molecular mechanisms. (Original content generated by AI) Overview of the mechanisms underlying bleomycin-induced pulmonary toxicity.

The immunopathogenesis of bleomycin-induced pulmonary toxicity remains incompletely defined and involves a convergence of oxidative cellular injury, dysregulated inflammatory cytokine signaling, and tissue-specific susceptibility related to low activity of the deactivating enzyme bleomycin hydrolase in lung tissue (**Figure 2**) (27, 28).

Bleomycin-induced pulmonary toxicity is initiated by direct cellular injury, wherein bleomycin generates iron-dependent reactive oxygen species that cause DNA strand breaks and oxidative injury to alveolar epithelial and endothelial cells (14). This injury leads to the release of damage-associated molecular patterns (DAMPs), endogenous signals that alert the innate immune system to tissue damage. These endogenous molecules subsequently trigger innate immune activation, resulting in sterile inflammation that reflects the host response to tissue injury (13). In experimental bleomycin lung injury, inflammatory mediator induction precedes cellular recruitment, with increased expression of proinflammatory cytokines, including TNF-α and IL-1β, alongside DAMP signaling activation such as high-mobility group box 1 (HMGB1), supporting the involvement of a DAMP-linked inflammatory cascade in this setting (13, 44). In line with this concept, innate pattern-recognition pathways have been implicated in bleomycin-associated fibrotic remodeling, with Toll-like receptor 4 (TLR4) signaling experimentally linked to promoting fibrosis in bleomycin-induced lung injury models (45).

Innate immune activation following bleomycin-induced injury appears to be mediated in part by alveolar macrophages, as they orchestrate cytokine release within the pulmonary tissue, including TNF-α, IL-1β, and IL-6. The release of these cytokines may amplify inflammatory signaling and contributes to sustained immune activation within the lung, while also promoting macrophage polarization toward a profibrotic phenotype (13, 14). A key consequence of IL-1 pathway activation is the induction of downstream cytokine axes that further amplify tissue remodeling. In particular, IL-17A has been identified as a potential mediator linking IL-1 signaling to fibrotic outcomes: in bleomycin-induced fibrosis, IL-17A is required for robust fibrotic remodeling in experimental systems, and an IL-1β/IL-23/IL-17 axis has been described as essential for pulmonary inflammation, remodeling, and fibrosis in the bleomycin model (46, 47).

Within the cytokine network activated by bleomycin-induced injury, transforming growth factor-β (TGF-β) is considered a central profibrotic mediator. TGF-β promotes fibroblast activation and extracellular matrix deposition, processes that contribute to the development of irreversible interstitial fibrosis (13, 14). Modern experimental work further supports that macrophage–fibroblast crosstalk is a major source of profibrotic signaling strength in bleomycin fibrosis, with monocyte-derived macrophage populations showing functional interactions with fibroblasts through canonical profibrotic pathways including TGF-β and PDGF (platelet-derived growth factor) (48). Together, these findings support a mechanistic framework in which initial bleomycin-mediated epithelial and endothelial injury is followed by DAMP-linked innate activation, macrophage-driven cytokine amplification (including IL-1 family and IL-17-related circuits), and subsequent engagement of profibrotic mediators such as TGF-β that promote fibroblast activation and extracellular matrix accumulation (13, 14, 44–48).

### 6.2 Risk factors for bleomycin-induced pulmonary toxicity

Despite increasing insight into the biological mechanisms of bleomycin-induced pulmonary toxicity, identifying patients at increased risk remains challenging. Multiple clinical and treatment-related risk factors for bleomycin-induced pulmonary toxicity have been described. Among these, cumulative bleomycin dose and renal function represent the most consistently identified and clinically relevant determinants of toxicity. Additional factors that have been investigated include advanced patient age, method of drug administration (particularly bolus dosing), smoking history or exposure to supplemental (8, 49). In clinical practice, cumulative tobacco exposure may be incorporated into individualized pulmonary risk assessment for bleomycin treatment, for example through measures reflecting both smoking intensity and duration rather than binary smoking status alone (50, 51).

The association between higher cumulative bleomycin exposure and the risk of bleomycin-induced pulmonary toxicity has been evaluated across multiple studies, although no universally accepted dose threshold has been established. Reported cutoff values associated with increased toxicity risk vary between studies. In patients with germ cell tumors, an elevated risk of bleomycin-induced pulmonary toxicity has been observed when cumulative doses exceed 300 mg, particularly in the presence of additional risk factors such as impaired renal function. In contrast, other analyses have identified cumulative doses above 240 mg as a principal risk factor for pulmonary toxicity (9, 52). This variability underscores the absence of a clear dose–toxicity threshold and highlights the importance of individualized risk assessment rather than reliance on a single cumulative dose limit. Importantly, the relationship between cumulative bleomycin exposure and clinically significant toxicity may also be influenced by treatment intensity and scheduling. In the GETUG-13 trial evaluating intensified chemotherapy strategies in poor-prognosis germ cell tumors, long-term follow-up demonstrated sustained therapeutic benefit without substantial persistent long-term toxicity attributable to bleomycin exposure, further illustrating the complexity of exposure–toxicity relationships in clinical practice (53).

Impaired renal function represents another major risk factor for the development of bleomycin-induced pulmonary toxicity, reflecting the predominant renal elimination of bleomycin. Since bleomycin is predominantly eliminated through the kidneys, concomitant cisplatin-associated renal impairment may further increase bleomycin exposure and toxicity risk (50). Consequently, reduced renal function may influence individualized selection among first-line cisplatin-based regimens, particularly when considering bleomycin-containing therapy (6, 50). Renal dysfunction is typically assessed using serum creatinine levels, creatinine clearance, and estimated glomerular filtration rate (GFR). In a cohort of patients with testicular cancer treated with bleomycin-containing chemotherapy, elevated serum creatinine levels were associated with a greater decline in diffusing capacity for carbon monoxide (DLCO), a functional marker of pulmonary toxicity (54). An increased risk of bleomycin-induced pulmonary toxicity has also been reported in patients with a baseline GFR below 80 mL/min prior to chemotherapy initiation. Advanced age has also been implicated as a risk factor for bleomycin-induced pulmonary toxicity, particularly in patients aged over 40 years at the time of bleomycin treatment (9).

Given the inflammatory features of bleomycin-induced pulmonary toxicity, the use of granulocyte colony-stimulating factor (G-CSF) has been examined as a potential risk modifier. The interaction between bleomycin and G-CSF, administered for the prevention of neutropenia and febrile neutropenia, has been investigated with conflicting results, with some studies reporting an increased risk of pulmonary toxicity (8, 49). However, other analyses have failed to confirm this association. In a comparative study of patients with germ cell tumors treated with bleomycin-containing regimens, no difference in the incidence of bleomycin-induced pulmonary toxicity was observed between patients receiving G-CSF and those who did not (55). Similarly, no adverse effect of filgrastim on pulmonary toxicity risk was identified in patients who developed bleomycin-induced pulmonary toxicity compared with those who did not (56). More recently, the use of pegfilgrastim in patients receiving bleomycin-containing chemotherapy was not associated with an increased incidence of bleomycin-induced pulmonary toxicity (57).

These observations indicate that although several risk factors for bleomycin-induced pulmonary toxicity have been described, their ability to predict toxicity in individual patients remains limited.

### 6.3 Measurement and assessment of bleomycin-induced pulmonary toxicity

Given the limited predictive value of clinical risk factors alone, accurate assessment of bleomycin-induced pulmonary toxicity is essential in the curative treatment of germ cell tumors, where early identification of lung injury may allow treatment modification and prevention of irreversible fibrosis. However, pulmonary toxicity remains challenging to detect at an early stage due to its heterogeneous clinical presentation, limited sensitivity of individual diagnostic modalities, and poor correlation between symptoms, imaging findings, and functional impairment, as consistently reported in clinical and survivorship studies (8, 9, 11).

Current approaches to pulmonary toxicity assessment rely on a combination of clinical evaluation, pulmonary function testing, imaging modalities, and emerging circulating biomarkers, each capturing distinct aspects of alveolar injury and immune-mediated inflammation (12, 13).

#### 6.3.1 Clinical assessment

Clinical manifestations of bleomycin pulmonary toxicity range from asymptomatic radiologic or functional abnormalities to rapidly progressive pneumonitis and respiratory failure (8). Common early symptoms include exertional dyspnea, non-productive cough, and reduced exercise tolerance, while fever and hypoxemia typically reflect more advanced inflammatory involvement (36). Importantly, clinical symptoms lack sensitivity for early lung injury and often appear only after substantial alveolar damage has occurred. Moreover, symptom severity correlates poorly with the extent of underlying inflammation or fibrosis, underscoring the limitations of symptom-based monitoring alone, particularly in young and otherwise fit patients treated with curative intent (9, 11). Clinical evaluation should incorporate baseline pulmonary comorbidity, cumulative tobacco exposure, and other factors potentially associated with increased pulmonary vulnerability, including anticipated exposure to supplemental oxygen or general anesthesia (6, 50, 51). In symptomatic patients, additional assessment of oxygenation status, including peripheral oxygen saturation or arterial blood gas analysis, may provide supportive clinical information.

#### 6.3.2 Pulmonary function testing

Pulmonary function tests (PFTs) represent the most widely used objective tool for monitoring bleomycin-related lung injury. Baseline pulmonary function assessment may include DLCO, forced vital capacity (FVC), forced expiratory volume in one second (FEV1), and the FEV1/FVC ratio providing broader characterization of baseline pulmonary function prior to bleomycin exposure (50, 58). Among PFT parameters DLCO is considered the most sensitive marker of early alveolar-capillary membrane damage and typically declines before changes in forced vital capacity (FVC) or total lung capacity (TLC), as demonstrated in both prospective studies and long-term follow-up of germ cell tumor survivors (11, 20).

Baseline and serial pulmonary function testing are frequently incorporated into clinical monitoring of patients receiving bleomycin-containing therapy, particularly with attention to DLCO trends over time (50). However, reductions in DLCO are non-specific and may be influenced by anemia, intercurrent infection, or pulmonary metastatic involvement. While serial DLCO monitoring can detect functional deterioration over time, its predictive value for clinically significant bleomycin pneumonitis remains limited, and no universally accepted DLCO threshold for treatment interruption or discontinuation has been established (8, 9). Consequently, PFT findings should be interpreted in conjunction with clinical presentation and imaging studies (50).

#### 6.3.3 Imaging modalities

High-resolution computed tomography (HRCT) is the imaging modality of choice for evaluating suspected bleomycin pulmonary toxicity. Typical radiologic findings include ground-glass opacities, reticular changes, and, in advanced cases, honeycombing and traction bronchiectasis, reflecting progressive inflammatory and fibrotic remodeling of the lung parenchyma (13, 27). Imaging abnormalities may precede overt clinical symptoms but often lag behind molecular and cellular immune activation occurring at the alveolar level. Conventional chest radiography lacks sensitivity for early toxicity and is insufficient as a standalone monitoring tool (8).

In this context, [^18F] fluorodeoxyglucose positron emission tomography (FDG-PET) has emerged as a potential adjunctive modality for detecting early inflammatory lung changes. Increased pulmonary FDG uptake reflects metabolically active inflammatory processes rather than established fibrosis and may therefore capture immune-mediated lung injury at a potentially reversible stage. In patients treated with bleomycin-containing regimens, diffuse or patchy pulmonary FDG uptake has been reported in the absence of overt clinical symptoms or significant structural abnormalities on CT, supporting the concept that FDG-PET may identify early immune activation within the lung parenchyma (59).

Nevertheless, FDG-PET findings are inherently non-specific and may be confounded by infection, tumor-related inflammation, or treatment-induced immune activation at other sites. Consequently, FDG-PET cannot be recommended as a routine screening tool for bleomycin pulmonary toxicity but may provide complementary information in selected high-risk patients or in cases with equivocal clinical, functional, or HRCT findings. Within an integrated assessment framework, FDG-PET should be regarded as a functional imaging surrogate of immune-mediated lung inflammation rather than a marker of irreversible fibrotic damage (59).

Thoracic ultrasonography has been investigated as a non-invasive imaging modality for the detection of bleomycin-induced pulmonary toxicity. In patients with clinically and radiologically confirmed toxicity, the number of comet tail artifacts on thoracic ultrasound was significantly increased and showed strong correlations with HRCT findings and reductions in pulmonary function parameters, particularly DLCO and FVC, with high diagnostic sensitivity and specificity. These findings suggest that thoracic ultrasonography may serve as a useful adjunct to established diagnostic tools for bleomycin-induced pulmonary toxicity, particularly in settings where repeated imaging or functional testing is limited (60).

Taken together, however, no single clinical or diagnostic modality reliably captures the full spectrum of bleomycin-induced pulmonary toxicity.

### 6.4 Biomarkers in predicting bleomycin-induced pulmonary toxicity

There have been limited biomarkers evaluated for the prediction of bleomycin-induced pulmonary toxicity. Bronchoalveolar lavage commonly demonstrates lymphocytosis, neutrophilia, and eosinophilia, alongside interstitial pneumonia and diffuse alveolar damage. In this context, lymphocyte-related inflammation markers have been shown to be prognostic for bleomycin-induced pulmonary toxicity in patients with testicular cancer (61). These observations suggest that while systemic inflammatory markers capture downstream immune activation, epithelial-derived biomarkers may provide a more direct window into alveolar injury and barrier disruption in bleomycin-induced pulmonary toxicity.

Accordingly, Krebs von den Lungen-6 (KL-6), a mucin-like glycoprotein released by injured or regenerating type II pneumocytes, has been investigated as a biomarker in interstitial lung disease and drug-induced pulmonary injury. Elevated circulating KL-6 levels have been associated with alveolar epithelial injury, impaired DLCO, and fibrotic remodeling across multiple ILD subtypes (62, 63). Experimental bleomycin-induced fibrosis models further support its biological relevance, as attenuation of fibrosis in IL-17A-deficient mice was accompanied by lower circulating KL-6 levels, consistent with reduced epithelial injury burden (64). At present, however, no circulating biomarker, including KL-6, has been prospectively validated for routine clinical decision-making in bleomycin pulmonary toxicity.

#### 6.4.1 The RAGE axis, SP-D and alveolar injury

The receptor for advanced glycation end products (RAGE) is highly expressed on type I alveolar epithelial cells within the lung. Soluble RAGE (sRAGE) reflects disruption of alveolar-immune homeostasis. In line with this, experimental models of acute lung injury have demonstrated increased RAGE concentrations in pulmonary edema fluid and bronchoalveolar lavage, a finding consistent with injury to type I alveolar cells and disruption of alveolar barrier integrity, with similar enrichment of RAGE in pulmonary edema fluid also observed in patients with acute lung injury (65, 66). Plasma RAGE has been demonstrated to be elevated in acute lung injury and ARDS, and in a randomized trial higher baseline levels measured in plasma samples were independently associated with greater severity of lung injury, increased mortality, and fewer ventilator-free and organ failure-free days (67). In parallel, studies evaluating soluble RAGE in plasma have shown that elevated baseline sRAGE concentrations in patients with ARDS are similarly associated with worse clinical outcomes, reinforcing its role as a circulating biomarker that integrates alveolar epithelial injury with functional and prognostic severity measures (68).

Surfactant protein D (SP-D) is predominantly synthesized by type II alveolar epithelial cells and contributes to pulmonary innate immune defense and regulation of inflammatory responses at the alveolar surface. During lung injury, disruption of the alveolar-capillary barrier permits leakage of SP-D from the airspaces into the systemic circulation, providing a mechanistic basis for its measurement as a circulatory biomarker of alveolar epithelial injury (69). In acute lung injury and ARDS, elevated plasma SP-D has demonstrated prognostic relevance. In a large randomized controlled trial, higher baseline plasma SP-D concentrations were independently associated with increased mortality, fewer ventilator-free days, and fewer organ failure-free days, supporting SP-D as an outcome-associated biomarker early in acute lung injury (70). In a retrospective analysis, plasma SP-D levels measured within 48 hours of ICU admission were higher in patients with ARDS compared to patients without ARDS and showed moderate diagnostic performance for ARDS identification (71). It has also been demonstrated that plasma SP-D levels increase under potentially injurious ventilator settings and are attenuated by lung-protective ventilation, whereas plasma sRAGE levels remain unchanged, indicating distinct biomarker dynamics in ventilator-associated lung injury (72). Beyond acute syndromes, serum SP-D has been reported to be elevated in idiopathic pulmonary fibrosis and associated with increased mortality risk, extending its biomarker relevance to chronic fibrotic lung disease (73).

#### 6.4.2 Translational implications for biomarker development

A multimodal approach integrating clinical evaluation, pulmonary function testing, structural and functional imaging, and emerging immune-based biomarkers may provide a more comprehensive strategy for early detection and risk stratification of patients at risk of developing bleomycin-induced pulmonary toxicity. Such an integrated framework would be particularly relevant in patients receiving bleomycin-containing regimens under curative intent, where prevention of irreversible lung injury is paramount (8, 9, 11).

Within this context, biomarkers represent promising tools for risk stratification and may enable individualized treatment selection and early intervention (61). Although the available evidence provides a strong biological rationale, much of the current evidence supporting SP-D and sRAGE has been derived from ARDS and other forms of acute lung injury, warranting cautious extrapolation to bleomycin-induced pulmonary toxicity. SP-D and sRAGE may capture key elements of bleomycin immunopathology by linking epithelial injury and immune activation. Importantly, their clinical relevance has been demonstrated in poor-risk GCT patients with choriocarcinoma syndrome, a severe form of acute respiratory failure after chemotherapy initiation. In this setting, elevated pretreatment SP-D and sRAGE levels were significantly associated with syndrome development, the need for mechanical ventilation, and inferior survival, whereas CC-16 was not predictive (74). These findings support SP-D and sRAGE as biologically plausible translational biomarkers of severe immune-mediated pulmonary injury. Nevertheless, they currently remain exploratory biomarkers, and their broader applicability to routine monitoring and risk stratification of bleomycin-induced pulmonary toxicity remains uncertain, because prospective validation in patients receiving bleomycin-containing first-line chemotherapy is still required before clinical implementation.

## 7 Conclusions

Bleomycin-induced pulmonary toxicity represents a paradigmatic example of immune-mediated chemotherapy toxicity in a curative oncologic setting. A more comprehensive understanding of its immunopathogenesis may improve recognition of early lung injury and refine individualized treatment decision-making in patients receiving bleomycin-containing therapy. At the same time, the translational integration of emerging biomarkers and immune-based risk stratification strategies remains an evolving field requiring further prospective validation.
